# DPV-001 an autophagosome-enriched cancer vaccine in phase II clinical trials contains 25 putative cancer antigens, DAMPS, HSPS and agonists for TLR 2, 3, 4, 7 and 9

**DOI:** 10.1186/2051-1426-1-S1-P260

**Published:** 2013-11-07

**Authors:** Traci L Hilton, Tyler W Hulett, Chistopher Dubay, Rieneke van de Ven, Sandra Aung, Walter J Urba, Hong Ming Hu, Bernard A Fox

**Affiliations:** 1 UbiVac, Portland, OR, USA; 2 Molecular Microbiology and Immunology, OHSU, Portland, OR, USA; 3 Laboratory of Molecular and Tumor Immunology, Robert W. Franz Cancer Research Center, Earle A. Chiles Research Institute, Providence Cancer Center, Portland, OR, USA; 4 Cancer Research, Robert W. Franz Cancer Research Center, Earle A. Chiles Research Institute, Providence Cancer Center, Portland, OR, USA; 5 Laboratory of Cancer Immunobiology, Robert W. Franz Cancer Research Center, Earle A. Chiles Research Institute, Providence Cancer Center, Portland, OR, USA

## 

Generation of a therapeutic immune response against a diverse set of antigens expressed by a patient's cancer is a central goal of cancer immunotherapy. This goal is important because diverse responses may prevent escape of antigen loss variants. To accomplish this goal we have developed tumor-derived autophagosome-enriched vaccines, "DRibbles", that sequester a complex mixture of proteins including cancer antigens and damage-associated molecular pattern molecules (DAMPs). In preclinical studies, combination immunotherapy with this vaccine approach provides significant protection from tumor challenge and therapeutic efficacy against established tumors. DRibble vaccines are superior to whole tumor vaccines in both protection and therapy studies, and demonstrated efficacy even when used across a complete MHC mismatch, opening the way for a clinical off-the-shelf cancer vaccine. UbiVac has produced an allogeneic DRibble vaccine (DPV-001) from two human NSCLC cell lines - UbiLT3 (non-specific histopathology) and UbiLT6 (adenocarcinoma-like) - that is currently used in a phase II trial for definitively treated stage IIIA/B NSCLC. Microarray analysis was performed on the cells at time of vaccine production and the vaccine was characterized by western blot, flow cytometry and liquid chromatography tandem mass spectrometry. Over 25 cancer-associated proteins have been identified in the DPV-001 vaccine including nine from the NCI's list of prioritized cancer antigens. Additionally found in this vaccine are multiple DAMPs, including S100A8, nucleolin, calreticulin, HMGB1, HSP70, HSP90, DNAs and RNAs. UbiLT3 DRibbles - but not whole cell irradiated vaccine - potently stimulates TLR 2, 3, 4, 7, and 9. Since many previously identified cancer antigens are created by genomic mutations and rearrangements, we performed exome sequencing of UbiLT3 and UbiLT6 and are searching for epitopes which could help break tolerance and improve vaccine efficacy. Together these data document that the DPV-001 vaccine contains antigens relevant for immunotherapy of cancer. In addition to NSCLC, we, and our collaborators, are exploring the application of this vaccine for the immunotherapy of colon, prostate and HNSCC.

